# Real world pharmacovigilance assessment of drug related perinatal depression risks

**DOI:** 10.3389/fphar.2026.1820606

**Published:** 2026-04-24

**Authors:** Jie-Hai Chen, Qing-Ming Luo, Yuan-Yan Tu

**Affiliations:** 1 Department of Anesthesiology, Dongguan Maternal and Child Healthcare Hospital, Dongguan, Guangdong, China; 2 Dongguan Maternal and Child Healthcare Hospital, Dongguan, Guangdong, China

**Keywords:** adverse drug events, disproportionality analysis, FAERS, perinatal depression, pharmacovigilance

## Abstract

**Background:**

Perinatal depression (PND) is a prevalent and serious mental health concern affecting women during pregnancy and the postpartum period. Although several drugs have been implicated in PND etiology in previous studies, large-scale systematic evaluations of drug-associated PND using real-world pharmacovigilance data remain scarce.

**Methods:**

We conducted a retrospective pharmacovigilance study utilizing the FDA Adverse Event Reporting System data spanning from Q1 2004 to Q2 2025. Disproportionality analysis, logistic regression, LASSO regression, and time-to-onset analysis were employed to examine drug-PND associations from multiple analytical perspectives.

**Results:**

Disproportionality analyses identified 28 drugs with positive signals as potentially associated with PND. Multi-factor analysis identified age under 26 years, weight exceeding 74 kg, and use of sodium oxybate, levonorgestrel, ethinylestradiol/etonogestrel, or lurasidone as independent risk factors for drug-related PND. The model achieved a ROC-AUC of 0.907, indicating excellent discriminatory performance.

**Conclusion:**

This study identified specific drugs and demographic factors associated with PND risk, offering valuable insights for pharmacovigilance and clinical medication management.

## 1 Introduction

Perinatal depression (PND) encompasses depressive episodes of varying severity occurring during pregnancy and up to 1 year after delivery, representing a critical public health concern (Al-abri et al., 2023; ACOG committee opinion, 2018). Affecting up to one in five individuals, it is among the most prevalent medical issues during pregnancy and the postpartum period (Clark et al., 2026). Despite its high prevalence, PND is often under-diagnosed and exerts enduring adverse consequences across the maternal-paternal-infant triad by eroding maternal quality of life, destabilizing couple intimacy, compromising obstetric outcomes and reducing breastfeeding likelihood, and programming adverse cognitive-emotional trajectories in offspring (Milgrom and Gemmill, 2014; Paulson et al., 2006; Letourneau et al., 2012). Multiple risk factors spanning social, biological, psychological, and genetic domains have been implicated in the etiology of PND (Dagher et al., 2021). Key risk factors include history of depression, stressful life events, unintended pregnancy, domestic violence, inadequate social support, traumatic birth experience, lower socioeconomic status, and drug abuse (ACOG committee opinion, 2018; Dagher et al., 2021; Nisar et al., 2020; Padhani et al., 2024; Thakuri et al., 2026).

Epidemiological research into the prevalence and associated factors of PND has continued to expand, with particular attention to modifiable risk factors that could inform preventive strategies (Al-abri et al., 2023; Padhani et al., 2024). Previous studies have identified several drug-related risk factors for PND, including synthetic oxytocin (Kroll-Desrosiers et al., 2017) and methyldopa (Wiciński et al., 2020). This underscores the multifactorial complexity of PND, in which drug-related contributors constitute a non-negligible component. However, drug-related PND has yet been fully explored in large-scale, systematic data. The identification of drug-induced PND signals in large-scale pharmacovigilance databases could inform regulatory decisions, guide clinical prescribing practices, and ultimately reduce the burden of iatrogenic depression in vulnerable perinatal populations.

The FDA Adverse Event Reporting System (FAERS) is the largest pharmacovigilance repository, aggregating spontaneous reports of adverse drug events to enable post-marketing surveillance of drug safety (Rodriguez et al., 2001). Therefore, this study sought to comprehensively evaluate the risk factors, signal characteristics, and onset patterns of drug-related PND. We aimed to delineate the drug-specific safety signals for postnatal depression, thereby furnishing evidence to guide therapeutic choices and mitigate medication-related PND.

## 2 Materials and methods

### 2.1 Data source

This retrospective pharmacovigilance study collected FAERS data covering the period from the first quarter (Q1) 2004 to Q2 2025. The publicly available data

Were downloaded from the FDA portal (https://fis.fda.gov/extensions/FPD-QDE-FAERS/FPD-QDE-FAERS.html). The database aggregates all spontaneously reported adverse events (AEs) from healthcare professionals, pharmaceutical manufacturers and consumers. FAERS data contained seven interlinked files: DEMO (patient demographic and administrative information), DRUG (suspect/concomitant medicines), REAC (adverse drug reactions), OUTC (patient outcomes), THER (drug therapy start and end dates), RPSR (report sources), and INDI (indications). To remove duplicate reports, we followed FDA-recommended deduplication logic: among entries sharing a CASEID we retained the latest FDA_DT; when both CASEID and FDA_DT were identical we preserved the highest PRIMARYID, ensuring each AE report was represented only once in the analytic set. PND reports were identified using the Medical Dictionary for Regulatory Activities (MedDRA) version 28.1 Preferred Term “perinatal depression” (PT code 10078366). Cases were restricted to those where the implicated drug was classified as the “primary suspect” for PND; reports listing the drug as “secondary suspect, concomitant, or interacting agent” were excluded.

### 2.2 Disproportionality analysis

In pharmacovigilance studies, disproportionality analyses were primarily applied to assess potential associations between specific drugs and particular AEs. We mined safety signals with a hybrid disproportionality framework that integrates frequentist and Bayesian frameworks: Reporting Odds Ratio (ROR) (Rothman et al., 2004), Proportional Reporting Ratio (PRR) (Evans et al.) with χ^2^ testing, Empirical Bayes Geometric Mean (EBGM) derived from the Multi-item Gamma-Poisson Shrinker (MGPS) (Heo and Jung, 2020), and Bayesian Confidence Propagation Neural Network (BCPNN) for Information Component (IC) estimation (Bate, 2007), thereby enhancing the robustness of detected safety signals. All four algorithms are based on 2 × 2 contingency tables (Supplementary Table S1). Computational expressions and signaling thresholds for all four disproportionality algorithms are detailed in Supplementary Table S2. Statistical significance was assessed after Bonferroni correction for multiple testing. P-adjust, defined as the p-value after Fisher’s exact test and Bonferroni correction, served as the threshold for significance when <0.01. To minimize false positives, only a signal satisfying all preset criteria for ROR, PRR, MGPS, and BCPNN was considered positive.

### 2.3 Regression analysis

We implemented a three-step modeling strategy to identify independent predictors of drug-induced PND. We first performed single-factor analysis for agents displaying significant ROR signals (case count >3, ROR lower bound of 95% CI > 1, and p-adjust <0.01). Second, potential candidates with p < 0.01 in these univariable models were advanced to the least absolute shrinkage and selection operator (LASSO) regression to mitigate multicollinearity and overfitting; only drugs retaining non-zero coefficients were retained for further analysis. Finally, we conducted multi-factor logistic regression model that incorporated the LASSO-selected drugs together with patient-level covariates (e.g., age, weight) as independent variables to determine the existence of drug-related PND risk factors. Only reports with complete age and weight information were included in multi-factor logistic regression.

To assess the final model’s predictive performance, we calculated the area under the receiver operating characteristic curve (AUC), wherein an AUC exceeding 0.7 indicates good discriminatory capability.

### 2.4 Time-to-onset analysis

The Time-to-onset (TTO) of adverse events was evaluated by computing the time interval between the event date (EVENT_DT) and the treatment start date (START_DT). To ensure logical consistency and completeness of data, records lacking complete dates or with EVENT_DT preceding START_DT were discarded. The Weibull shape parameter (WSP) test is used to analyze TTO data of drugs identified as independent risk factors in the multivariate logistic regression (Ogura and Shiraishi, 2023). One additional day was incorporated into the calculation, as the drug initiation date was considered as day 1. The Weibull scale parameter (α) regulates the distributional dispersion, while the shape parameter (β) intrinsically defines the hazard trajectory. Interpretation of failure rates enables classification of adverse event patterns: β < 1 with 95% CI < 1 indicates temporally decreasing hazard (early failure); β ≈ 1 with 95% CI containing one signifies time-invariant hazard (random failure); β > 1 with 95% CI excluding one demonstrates hazard maximization at a specific temporal point (wear-out failure).

### 2.5 Statistical analysis

We used descriptive statistics to profile the clinical features of patients with drug-associated PND reports. Potential independent predictors were identified with multivariable logistic regression followed by Bonferroni adjustment for multiple comparisons. The threshold for significance was set at a Bonferroni-corrected p-value (p-adjust) of <0.01. This investigation was carried out in compliance with the reporting of a disproportionality analysis for drug safety signal detection using individual case safety reports in pharmacovigilance (READUS-PV) (Fusaroli et al., 2024) (Supplementary Table S3). All Data processing and statistical analysis were carried out in R version 4.3.3.

## 3 Results

### 3.1 Baseline characteristics of PND

**FIGURE 1:**
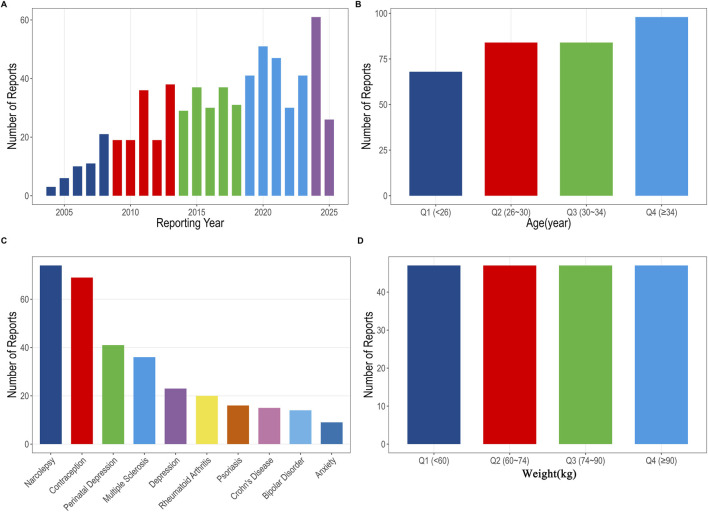
Overview of drug-related PND reports. **(A)** Drug-related PND reports by year; **(B)** Drug-related PND reports by patient age; **(C)** Primary diagnoses of patients with drug-related PND; **(D)** Drug-related PND reports by patient weight. PND, perinatal depression.

During the period from Q1 2004 to Q2 2025, the FAERS database recorded a total of 18, 937, 868 (deduplicated) AE reports, of which 643 were PND-related cases. The baseline characteristics of drug-related PND are shown in Table 1 and Figure 1. Regarding gender distribution, female patients predominated, accounting for 94.4% of cases. There were just four recorded male cases, and 32 reports (5%) lacked gender information. The median age and weight of the patients were 30 years (interquartile range [IQR] 26–34 years) and 74.4 kg (IQR 60–90 kg), respectively. Temporal analysis revealed that the number of PND-related reports increased steadily over the study period, reaching a peak of 61 cases in 2024 (Figure 1A). Age-stratified analysis revealed that patients aged >34 years had the highest absolute number of drug-related PND reports (Figure 1B); this may reflect higher medication utilization in this age group rather than increased susceptibility. However, the reports were fairly evenly spread across different weight groups (Figure 1D). Regarding the diagnostic backgrounds of the reported cases, PND was most commonly associated with narcolepsy, contraception, and perinatal depression (Figure 1C).

**TABLE 1:** Baseline information of drug-induced PND.

Characteristics	Drug-related PND (N = 643)
Age	
<10	3 (0.5%)
10–26	82 (12.8%)
26–34	175 (27.2%)
34–50	64 (10%)
>50	10 (1.6%)
Unknown	309 (48.1%)
Gender	
Female	607 (94.4%)
Male	4 (0.6%)
Unknown	32 (5.0%)
Weight	
Median (Q1, Q3)	74.4 (60, 90)
Unknown	455 (70.8%)
Occupation of the reporter	
Physician	126 (19.6%)
Pharmacist	11 (1.7%)
Other health-professional	110 (17.1%)
Lawyer	21 (3.3%)
Consumer	339 (52.7%)
Unknown	36 (5.6%)
Reported country (top 5)	
United States	446 (69.4%)
Canada	35 (5.4%)
United Kingdom	29 (4.5%)
Germany	22 (3.4%)
Brazil	18 (2.8%)

### 3.2 Drugs associated with PND

From an initial screening of 147 unique drugs, agents with fewer than three reported cases were excluded. Four signal detection algorithms (ROR, PRR, MGPS, and BCPNN) identified 28 drugs with positive signals as potentially associated with PND (Supplementary Table S4). The top five drug categories involved in drug-induced PND were contraceptives (6/28), antidepressants (6/28), agents for postpartum depression (2/28), antipsychotics (2/28), and antiepileptics (2/28).

At the individual drug level, the top 10 drugs by incident frequency of PND events reported were: gamma hydroxybutyrate (a = 81, ROR = 39.23), levonorgestrel (a = 43, ROR = 7.08), certolizumab pegol (a = 23, ROR = 8.08), zuranolone (a = 19, ROR = 933.39), etonogestrel (a = 18, ROR = 10.16), interferon beta 1a (a = 17, ROR = 3.31), sertraline (a = 16, ROR = 9.14), brexanolone (a = 15, ROR = 2025.67), ethinylestradiol/etonogestrel (a = 15, ROR = 24.02), and quetiapine (a = 14, ROR = 5.40).

**FIGURE 2:**
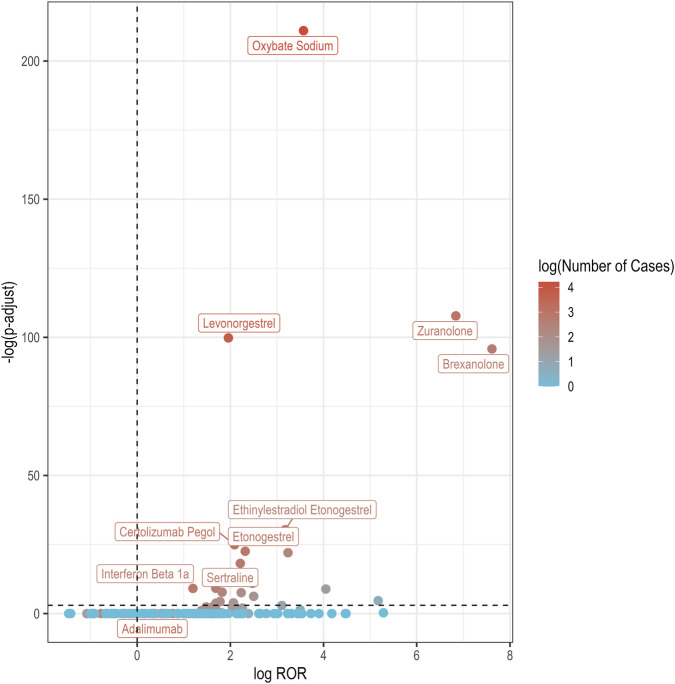
PND-related drug volcano plots. PND, perinatal depression; ROR, reporting odds ratio; p-adjust, p-value after Bonferroni correction.

To visualize signal strengths, a volcano plot presents the relationship between these suspected drugs and PND (Figure 2). In this visualization, the x-axis displays the logarithmic transformation of ROR, where positive values denote a higher reporting frequency of PND-related adverse events compared to other adverse reactions. The y-axis shows the negative logarithm of p-adjust, derived from Fisher’s exact test with Bonferroni correction, with positive values indicating greater statistical significance. Dot color represents the logarithm of case report counts, with redder hues corresponding to larger report volumes. Consequently, drugs positioned in the upper-right quadrant exhibited both robust signal strength and statistically significant differences.

### 3.3 Risk factors for drug-related PND

To identify independent predictors of drug-related PND beyond crude disproportionality signals, we implemented a rigorous three-step modeling approach integrating univariable screening, LASSO regularization, and mul-tivariable logistic regression.

**FIGURE 3:**
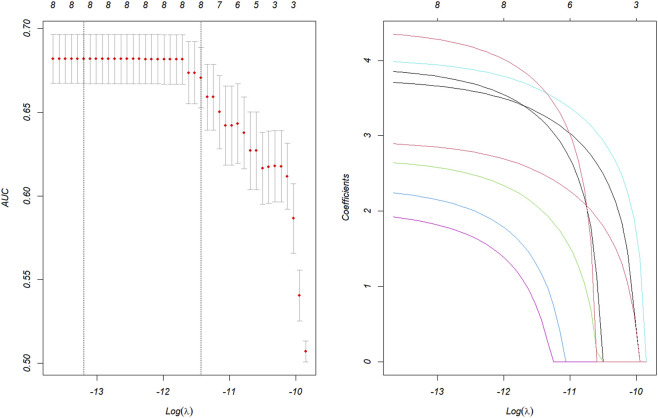
Results of the LASSO regression analysis. LASSO, least absolute shrinkage and selection operator.

**FIGURE 4:**
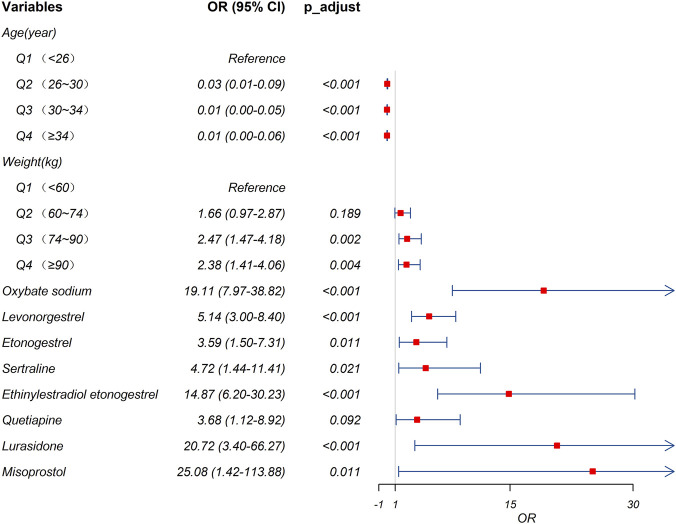
Results of the multi-factor logistic regression analysis. CI, confidence interval; OR, odds ratio; p-adjust, p-value after Bonferroni correction. A p-adjust value <0.01 indicates statistical significance.

**FIGURE 5:**
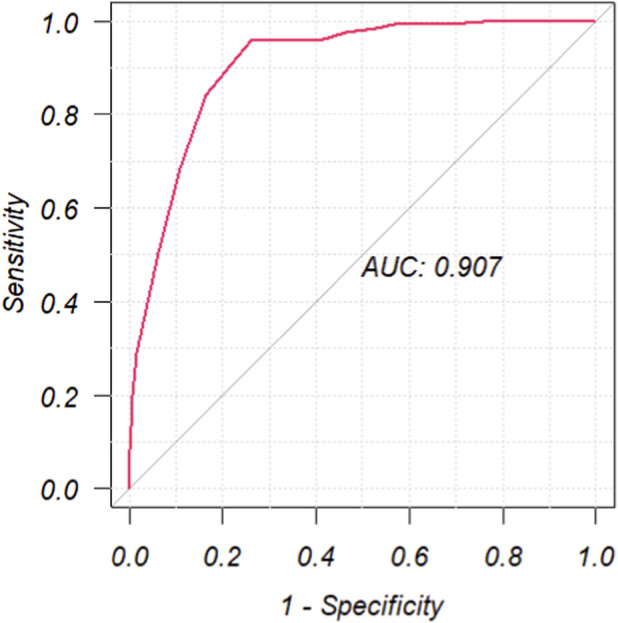
The ROC curves of drug-related PND risk factors. PND, perinatal depression; ROC, receiver operating characteristic; AUC, area under curve. An AUC greater than 0.7 indicates good performance.

Candidate drugs having more than three case reports, a lower 95% CI limit of the ROR >1, and p-adjust <0.01 were selected for single-factor analysis. A total of eight medications were tested using LASSO regression for medications with p < 0.01 in univariate analysis (Figure 3). Subsequently, these drugs were subjected to multi-factor logistic regression analysis in conjunction with patient demographic characteristics such as age and weight. The findings revealed that age under 26 years old, weight greater than 74 kg, and four drugs, including oxybate sodium, levonorgestrel, ethinylestradiol/etonogestrel and lurasidone, were independent risk factors for drug-related PND (Figure 4). The model demonstrated favorable predictive capability for drug-induced PND risk based on the selected variables, achieving a ROC-AUC of 0.907 (Figure 5). It is important to note that this AUC reflects predictive performance and does not imply causation; the identified associations should not be interpreted as causal effects without further validation in independent cohorts.

### 3.4 Time-to-onset of drug-induced PND

**FIGURE 6:**
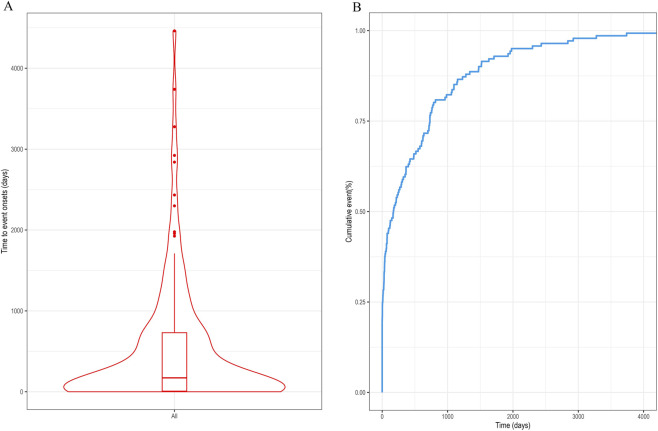
Onset time analysis of PND-related drugs. **(A)** Violin plot of time to drug-related PND occurrence; **(B)** Cumulative incidence of PND-related adverse events across all drugs. PND, perinatal depression.

The Time-to-onset (TTO) of drug-induced PND was evaluated (Figure 6). The median TTO of 172 days (IQR 8–730 days) indicates substantial variability in the temporal presentation of drug-induced PND. The observation that nearly 50% of cases emerged within 180 days suggests a critical monitoring window during the first 6 months of therapy. However, the wide interquartile range (extending to 730 days) underscores the need for sustained vigilance throughout the perinatal period and beyond. Table 2 presents the WSP analysis for the drugs identified as independent risk factors in the multivariate regression analysis. As there are numerous missing values in the TTO data, these results should be interpreted with caution.

**TABLE 2:** Time-to-onset analysis for specific PND-related drugs.

Drug*	Cases (n)	TTO (days)		Weibull distribution				Failure type
				Scale parameter		Shape parameter		
		Median (IQR)	Min ∼ Max	α	95% CI	β	95% CI	
Levonorgestrel	17	215.0 (1.0–731.0)	1–1,521	210	−37.38–457.38	0.42	0.25–0.6	Early failure
Ethinylestradiol etonogestrel	6	106.5 (74.0–496.8)	52–3,277	458.02	−165.21–1,081.24	0.63	0.26–0.99	Early failure

n, number of cases with available time-to-onset; IQR, interquartile range; TTO, Time-to-onset. *Weibull shape parameter analysis could not be performed for oxybate sodium and lurasidone due to insufficient valid TTO, data.

## 4 Discussion

This large-scale pharmacovigilance analysis represents the most comprehensive evaluation of drug-related PND risk factors to date. By integrating multiple analytical methodologies—disproportionality analysis, LASSO regression, and multivariable regression—we provide robust, convergent evidence identifying specific demographic and pharmacological risk factors for PND. This comprehensive analysis provides evidence that may inform clinical risk stratification, contribute to pharmacovigilance signal refinement, and generate hypotheses for future mechanistic studies.

Perinatal depression encompasses non-psychotic depressive episodes occurring during pregnancy and extending through the first postpartum year, a critical window characterized by the peak incidence of maternal mental health disorders with substantial adverse effects on both maternal and infant wellbeing (Al-abri et al., 2023; Stevenson et al., 2023). The global health literature emphasizes the fundamental importance of mental wellbeing, as underscored by the maxims “no health without mental health” (Prince et al., 2007) and “no health without perinatal mental health” (Howard et al., 2014). In recent years, a growing body of research has addressed this global health concern, focusing on investigating the incidence, risk factors, and etiology of PND (Al-abri et al., 2023; Dagher et al., 2021; Thakuri et al., 2026; Gastaldon et al., 2022). Certain medications such as synthetic oxytocin (Kroll-Desrosiers et al., 2017) and methyldopa (Wiciński et al., 2020), are also considered risk factors for inducing PND. The rarity of drug-induced PND poses considerable challenges in establishing specific medications as causative agents for this condition. Consequently, a large-scale investigation are warranted to identify suspect medications and elucidate critical risk factors associated with drug-related PND.

We utilized FAERS, the world’s largest spontaneous reporting system for adverse drug events, to comprehensively investigate suspected drugs and risk factors associated with PND. During the study period, our study observed the median age of drug-related PND patients was 30 years (IQR 26–34 years), which aligns broadly with findings from prior Swedish nationwide investigations of PND. Hagatulah et al. (Hagatulah et al., 2024)reported a median age of 31 years (IQR 27–37 years) among 86,551 patients with PND in Sweden. Furthermore, our study identified being under 26 years of age as an independent risk factor for drug-related PND, whilst older age demonstrated a protective effect. Consistent with our findings, a previous descriptive cross-sectional study (Seidi et al., 2022) identified a statistically significant association between perinatal depressive symptoms and younger age, with older age exhibiting a protective effect against such symptoms. Similarly, Ronen et al. (Ronen et al., 2024) noted that young mothers aged <25 years face a higher risk of PND, which may help explain our findings. Healthcare providers managing pregnant and postpartum women—particularly those prescribing medications to individuals under 26 years—should implement enhanced screening protocols and patient education regarding PND risk. The identification of young maternal age as an independent risk factor, even after controlling for specific drug exposures, suggests that developmental and psychosocial factors may confer heightened vulnerability to pharmacologically-induced mood disturbances.

Regarding body weight, the median value among reported PND patients was 74.4 kg (IQR 60–90 kg). Multivariate logistic regression analysis further identified body weight >74 kg as an independent risk factor, consistent with prior evidence establishing maternal obesity is an independent risk factor for PND. A retrospective cohort study (de Los Reyes et al., 2023) demonstrated that obesity confers an elevated risk of perinatal depression compared with normal weight women, underscoring the significance of maintaining healthy body weight. Similarly, a multicenter study conducted by Ventriglio et al. (Ventriglio et al., 2023) found that overweight and obesity (higher levels of BMI) were associated with a higher risk of PND. The putative mechanisms underlying the obesity-PND association encompass dysregulation of the hypothalamic-pituitary-adrenal (HPA) axis, a common feature in both conditions, along with elevated circulating glucocorticoids, heightened inflammation markers, increased oxidative stress, gut microbiome alterations, and psychological distress related to body image disturbance, all of which have been extensively discussed in the literature (Lopresti et al., 2013; Bernabé et al., 2019; Avalos et al., 2020).

It is noteworthy that synthetic oxytocin (Kroll-Desrosiers et al., 2017) and methyldopa (Wiciński et al., 2020), previously implicated in PND, were not identified as significant signals in the present analysis. This discrepancy likely stems from inherent limitations of the FAERS database: both drugs had fewer than three reports with PND as the primary suspect, falling below the minimum threshold required for inclusion in our disproportionality analysis. This absence does not negate a potential relationship but rather reflects that spontaneous reporting systems may under-represent certain drug-event associations due to underreporting, differences in prescription frequency, or variations in clinical awareness. Therefore, the lack of detection for these two drugs should be interpreted with caution and does not diminish the validity of previously reported associations; rather, it underscores the complementary roles of spontaneous reporting systems and targeted clinical studies in comprehensive drug safety surveillance.

Suspected drugs in our study were selected based on > 3 case reports, ROR lower 95% CI > 1, and Bonferroni-corrected p-value <0.01. Subsequent screening incorporated single-factor analysis, LASSO regression, and multi-factor logistic regression, ultimately identifying four drugs as independent risk factors for drug-related PND: sodium oxybate, levonorgestrel, ethinylestradiol/etonogestrel, and lurasidone. Gamma-hydroxybutyrate (GHB, administered as sodium oxybate), an endogenous short-chain fatty acid in the brain, is therapeutically employed for narcolepsy and alcohol abuse/withdrawal. There have been reports of depression following withdrawal of sodium oxybate, and of sodium oxybate being used as a potential antidepressant (Domínguez et al., 2015; Mamelak, 2009). However, the relationship between sodium oxybate and PND has been scarcely elucidated in literature. Our finding of sodium oxybate as an independent PND risk factor suggests that its effects on perinatal mood may be context-dependent, potentially influenced by hormonal milieu, sleep architecture alterations, or withdrawal phenomena. In our study, contraception emerged as one of the most frequent indications among patients with drug-related PND (Figure 1C), with contraceptives representing the predominant drug category implicated in drug-induced PND (Supplementary Table S4). Levonorgestrel and ethinylestradiol/etonogestrel are widely prescribed as contraceptives and are well tolerated. A nationwide prospective cohort study in Denmark involving 1,061,997 women demonstrated that hormonal contraceptives such as levonorgestrel and etonogestrel are associated with a higher risk of depression (Skovlund et al., 2016). A longitudinal cohort study involving 703,157 women in Sweden indicates that levonorgestrel users are at increased risk of developing depression (Stenhammar et al., 2023). The association between progestogen-containing contraceptives and depression has been further substantiated by recent evidence. Gao et al. (Gao et al., 2025) conducted a comprehensive FAERS-based pharmacovigilance study and reported positive signals for levonorgestrel, etonogestrel, and desogestrel in relation to depression. Moreover, a nationwide prospective cohort study from Denmark by Skovlund et al. (Skovlund et al., 2024)demonstrated a dose-dependent association between levonorgestrel-releasing intrauterine systems and incident depression risk. These studies support our findings and align with the theory that progesterone plays a role in the etiology of depression. Our study also identified lurasidone as a risk factor for drug-related PND (OR 20.72; 95% CI 3.40–66.27). Lurasidone, a second-generation antipsychotic, is indicated for the management of schizophrenia and bipolar depression. Several mechanisms could explain this apparent paradox: (1) confounding by indication if lurasidone was prescribed for pre-existing mood instability that subsequently progressed to PND; (2) pharmacokinetic interactions with pregnancy-related physiological changes; or (3) differential effects of lurasidone on dopamine D2, serotonin 5-HT7, and 5-HT1A receptors in the context of perinatal neuroplasticity. This signal warrants investigation through clinical studies and mechanistic research.

We observed substantial variability in the Time-to-onset (TTO) of drug-associated PND (IQR: 8–730 days). This heterogeneity may be attributable to: inter-drug differences in pharmacokinetic properties and mechanisms of action; inconsistent definitions and recognition of PND onset among reporters; and potential recall bias inherent in spontaneous reporting systems. The median TTO of 172 days suggests that monitoring for depressive symptoms should extend well beyond the initial weeks of treatment. Clinicians prescribing these medications to perinatal individuals should consider baseline depression screening and periodic follow-up, especially in high-risk subgroups.

This study provided a comprehensive overview of the potential risks associated with drug-related PND through large-scale, systematic data. However, some limitations of FAERS data must be acknowledged. Firstly, as a spontaneous reporting system, FAERS is dependent on voluntary submissions, which may result in underreporting, duplicate entries, and data inaccuracies, thereby potentially compromising data reliability. Secondly, the absence of data on healthy populations precluded the calculation of drug-related PND incidence. Thirdly, while FAERS contains variables such as geographic location and reporter occupation, these were not included as covariates in our multivariable models. Geographic information is often incomplete or recorded at a coarse level, limiting its utility as a proxy for population-level genetic variability. Reporter occupation primarily reflects reporting source rather than biological risk, and its inclusion could introduce over-adjustment for reporting bias. Nonetheless, we acknowledge that these factors may influence signal detection, and future studies with more granular data are warranted to explore their potential impact. Finally, although disproportionality analysis is valuable for detecting potential drug-event associations, it does not infer causality. Our findings should be considered hypothesis-generating, and causal inference requires confirmation through well-designed prospective studies. Despite these limitations, this study represents one of the most extensive pharmacovigilance analyses of drug-induced PND utilizing FAERS data. Our multi-method approach strengthens signal robustness and provides a foundation for subsequent epidemiological investigations and clinical trials. Although FAERS data alone are insufficient to establish causality, this research yields valuable insights for refining pharmacovigilance policies and enhancing clinical decision-making.

## 5 Conclusion

In summary, this study leveraged real-world adverse drug event data to comprehensively evaluate the risk factors of drug-related PND. Our analysis revealed that age under 26 years, weight exceeding 74 kg, and use of sodium oxybate, levonorgestrel, ethinylestradiol/etonogestrel, or lurasidone were independently associated with drug-induced PND. These findings highlight the importance of vigilance when prescribing these medications, providing real-world evidence to inform mechanistic investigations and strengthen pharmacovigilance strategies.

## Data Availability

The original contributions presented in the study are included in the article/Supplementary Material, further inquiries can be directed to the corresponding authors.
