# Supplementary material for: Real world pharmacovigilance assessment of drug related perinatal depression risks
Source: Front Pharmacol. 2026 Apr 24;17:1820606. doi: 10.3389/fphar.2026.1820606 (PMC13153133; doi:10.3389/fphar.2026.1820606)
Supplement: Supplementary file 1 [file Supplementaryfile1.docx]

Supplementary Table S1. Two-by-two contingency table for disproportionality analysis.

| Item | Target adverse events reported | Other adverse events reported | Total |
| --- | --- | --- | --- |
| Target drugs | a | b | a + b |
| Other drugs | c | d | c + d |
| Total | a + c | b + d | a + b + c + d |

Supplementary Table S2. Four major algorithms used for signal detection.

| Method | Calculation formula | ﻿Criteria |
| --- | --- | --- |
| ROR | $ROR=\frac{a / c}{b / d}$ | a ≥ 3  95%CI (lower limit) > 1 |
|  | $SE(lnROR)=\sqrt{\frac{1}{a}+\frac{1}{b}+\frac{1}{c}+\frac{1}{d}}$ |  |
|  | $95\%CI= e^{\ln\left( ROR \right)\pm1.96se}$ |  |
| PRR | $PRR=\frac{a / (a+b)}{c / (c+d)}$ | a ≥ 3  PRR ≥ 2  $\chi2\geq4$ |
|  | $\chi2 =\frac{{(ad-bc)}^{2}(a+b+c+d)}{( a+b)(a+c)(c+d)(b+d)}$ |  |
| BCPNN | IC=${log}_{2}\frac{p(x,y)}{p(x)p(y)}={log}_{2}\frac{a(a+b+c+d)}{(a+b)(a+c)}$ | IC025>0 |
|  | E(IC)=${log}_{2}\frac{(a+\gamma11)(a+b+c+d+\alpha)(a+b+c+d+\beta)}{（a+b+c+d+\gamma）(a+b+\alpha1)(a+c+\beta1)}$ |  |
|  | $V\left( IC \right)=\frac{1}{{(ln2)}^{2}}\{\left[ \frac{\left( a+b+c+d \right)-a+\gamma-\gamma11}{\left( a+\gamma11 \right)\left( 1+a+b+c+d+\gamma\right)} \right]+\left[ \frac{\left( a+b+c+d \right)-\left( a+b \right)+\alpha-\alpha1}{\left( a+b+\alpha1 \right)\left( 1+a+b+c+d+\alpha\right)} \right]+\left[ \frac{\left( a+b+c+d \right)-\left( a+c \right)+\beta-\beta1}{\left( a+c+\beta1 \right)\left( 1+a+b+c+d+\beta\right)} \right]\}$ |  |
|  | $\gamma=\gamma11\frac{(a+b+c+d+\alpha)(a+b+c+d+\beta)}{(a+b+\alpha1)(a+c+\beta1)}$ |  |
|  | *IC-2SD=E(IC)-2*$\sqrt{V(IC)}$  $\alpha1=\beta1=1；\alpha=\beta=2；\gamma11=1$ |  |
| EBGM | $EBGM=\frac{a(a+b+c+d)}{\left( a+c \right)(a+b)}$ | EBGM05>2 |
|  | $SE(lnEBGM)=\sqrt{\frac{1}{a}+\frac{1}{b}+\frac{1}{c}+\frac{1}{d}}$ |  |
|  | $95\%CI= e^{\ln\left( EBGM \right)\pm1.96se}$ |  |

Equation: a, number of reports containing both the suspect drug and the suspect adverse drug reaction; b, number of reports containing the suspect adverse drug reaction with other medications (except the drug of interest); c, number of reports containing the suspect drug with other adverse drug reactions (except the event of interest); d, number of reports containing other medications and other adverse drug reactions. ROR, reporting odds ratio; CI, confidence interval; N, the number of co-occurrences; PRR, proportional reporting ratio; χ2, chi-squared; BCPNN, Bayesian confidence propagation neural network; IC, information component; IC025, the lower limit of the 95% one-sided CI of the IC; EBGM: Empirical Bayes Geometric Mean; EBGM05, The lower limit of the 90% one-sided CI of the EBGM.

Supplementary Table S3. The READUS-PV checklist

| **Section and topic** | **Item #** | **Checklist item** | **Location where item is reported** |
| --- | --- | --- | --- |
| **Title** |  |  |  |
|  | *1a* | If disproportionality analyses are a prominent component of the published study, the study should be identified as a “disproportionality analysis”. The type of data and name of the database(s) should be specified. | Page 1 |
|  | *1b* | Report the name of adverse event(s) and/or drug(s) under study, when applicable. | Page 1 |
| **Introduction** |  |  |  |
| Background | *2a* | Describe the drug(s) and its utilization, the nature of the adverse event(s) under study and its frequency, and the existing knowledge on the drug-event combination. | Page 3-4 |
|  | *2b* | Specify the rationale for performing the analysis, e.g., as part of routine pharmacovigilance, to investigate an overall safety profile, or to assess a pre-specified hypothesis. | Page 3-4 |
|  | *2c* | Explain why ICSR databases and disproportionality analysis are suitable to fill the knowledge gap. | Page 4 |
| Objectives | *3* | State specific objectives, identifying the adverse event(s), the drug(s), and the reference group, including any pre-specified hypothesis, if applicable. | Page 4 |
| **Methods** |  |  |  |
| Study design | *4a* | Identify the study (i.e., “disproportionality analysis”) and the type of data used (e.g., “individual case safety reports”). | Page 4-5 |
|  | *4b* | Provide an outline of the entire study design, including primary and sensitivity analyses performed, and other designs such as case-by-case analysis or literature review. | Page 4-5 |
| Data description, access, and pre-processing | *5a* | Specify the name of the database(s), the database(s) custodian, and the coverage. Specify the type/number of drugs included within the database and the thesaurus, taxonomies, or ontologies used for coding drugs and events. | Page 4-5 |
|  | *5b* | Specify the extraction dates and describe and justify all choices used for data pre-processing, including any data transformation or exclusion, if appropriate. | Page 4-5 |
| Variables definition | *6a* | Describe the study population, including any restriction. | Page 4-5 |
|  | *6b* | Describe the nature and the meaning of key variables assessed in the work. | Page 4-5 |
|  | *6c* | Specify and justify any grouping of drugs or events. For drugs, specify and justify whether active ingredients/trade names/salts were considered and/or the selected role. | Page 5 |
|  | *6d* | Describe any additional data source used, the type of data, and how they interact with ICSRs. | Not applicable |
| Statistical methods | *7a* | Present any descriptive analysis performed, specifying variables investigated, statistical tests, and significance thresholds. | Page 6-7 |
|  | *7b* | Describe the measure(s) selected for the disproportionality analysis including any threshold used to identify signals of disproportionate reporting. Explain the reason for this choice if applicable. | Page 6-7 |
|  | *7c* | Clearly describe any sensitivity analysis and any tool to control confounding, including any restriction, subgroup, stratification, adjustment, or interaction. | Page 6-7 |
|  | *7d* | Specify the variables and methods used for the case-by-case analysis, including any algorithm or criteria used to assess causality, if performed. | Not applicable |
|  | *7e* | Specify any statistical methods used for other data sources. | Not applicable |
| **Results** |  |  |  |
| Participants | *8a* | Specify the number of individual case safety reports included at each stage, including reasons for exclusion. | Page 8 |
|  | *8b* | Provide key demographic and clinical characteristics of cases, if possible comparing cases with any appropriate reference group. | Page 8 |
| Disproportionality analysis | *9* | Present all results including confidence intervals. Present also results of sensitivity analyses, if performed. | Page 9 |
| Case-by-case analysis | *10* | Present the case-by-case analysis of key variables. Present the causality assessment, if applicable. | Page 8-9 |
| **Discussion** |  |  |  |
| Key results | *11* | Discuss key results with reference to study objectives and contextualize them within the current literature and other consulted sources. Clearly discriminate between expected reactions and emerging safety signals. | Page 11-14 |
| External validity | *12a* | Discuss the external validity of the results to the general population. | Page 11-16 |
|  | *12b* | Discuss the potential relevance of results in clinical practice | Page 11-16 |
|  | *12c* | Propose further study designs if applicable | Not applicable |
| Limitations | *13* | Present general limitations, making clear that disproportionality analysis alone cannot prove causation or measure incidence, and specific limitations, including confounding and reporting bias and efforts to mitigate them. | Page 16 |
| **Declarations** |  |  |  |
|  | *14a* | Provide the source of funding/sponsorship and the role of the funders/sponsors for the present study and for any original study on which the present article is based. | Page 18 |
|  | *14b* | Clearly identify potential commercial and intellectual conflicts of interest (e.g., link to any drug/event investigated, whether financial, legal action, or software used). | Page 18 |
|  | *14c* | Declare any institutional approval needed or granted in the investigation. | Page 17 |
|  | *14d* | Include a statement on data availability, code availability (including the version of the statistical software used), and protocol registration. | Page 18 |

Supplementary Table S4. PND-related drugs reported in FAERS.

| **Drug Category** | **Drug** | **a** | **ROR(95%Cl)** | **PRR(χ^2^)** | **EBGM(EBGM05)** | **IC(IC025)** | **pvalue** | **p_adjust** |
| --- | --- | --- | --- | --- | --- | --- | --- | --- |
| Agent for postpartum depression | zuranolone | 19 | 933.39 ( 587.41 - 1483.15 ) | 906.01 ( 16669.24 ) | 879.27 ( 553.35 ) | 9.78 ( 3.63 ) | 1.08E-49 | 1.58E-47 |
| Agent for postpartum depression | brexanolone | 15 | 2025.67 ( 1194.3 - 3435.79 ) | 1900.18 ( 27809.38 ) | 1855.87 ( 1094.19 ) | 10.86 ( 3.23 ) | 1.70E-44 | 2.50E-42 |
| Antiepileptic | topiramate | 9 | 11.88 ( 6.15 - 22.95 ) | 11.88 ( 88.41 ) | 11.73 ( 6.07 ) | 3.55 ( 1.58 ) | 1.22E-07 | 1.79E-05 |
| Antiepileptic | levetiracetam | 8 | 4.4 ( 2.19 - 8.83 ) | 4.4 ( 20.74 ) | 4.36 ( 2.17 ) | 2.12 ( 0.7 ) | 6.23E-04 | 0.091567508 |
| Antidepressant | sertraline | 16 | 9.14 ( 5.56 - 15.01 ) | 9.13 ( 113.01 ) | 8.93 ( 5.44 ) | 3.16 ( 1.9 ) | 8.62E-11 | 1.27E-08 |
| Antidepressant | duloxetine | 11 | 6.19 ( 3.41 - 11.24 ) | 6.19 ( 47.06 ) | 6.1 ( 3.36 ) | 2.61 ( 1.26 ) | 2.98E-06 | 4.38E-04 |
| Antidepressant | fluoxetine | 8 | 9.32 ( 4.64 - 18.73 ) | 9.32 ( 58.69 ) | 9.22 ( 4.59 ) | 3.2 ( 1.3 ) | 3.58E-06 | 5.26E-04 |
| Antidepressant | bupropion | 7 | 5.08 ( 2.41 - 10.71 ) | 5.08 ( 22.71 ) | 5.04 ( 2.39 ) | 2.33 ( 0.72 ) | 5.82E-04 | 0.085599593 |
| Antidepressant | paroxetine | 7 | 5.37 ( 2.55 - 11.31 ) | 5.37 ( 24.6 ) | 5.32 ( 2.53 ) | 2.41 ( 0.76 ) | 4.24E-04 | 0.062382543 |
| Antidepressant | escitalopram | 6 | 7.9 ( 3.54 - 17.65 ) | 7.9 ( 35.81 ) | 7.83 ( 3.51 ) | 2.97 ( 0.89 ) | 1.44E-04 | 0.02109761 |
| Antiemetic | ondansetron | 11 | 25.48 ( 14.04 - 46.26 ) | 25.46 ( 254.09 ) | 25.04 ( 13.79 ) | 4.65 ( 2.22 ) | 1.81E-12 | 2.66E-10 |
| Antipsychotic | quetiapine | 14 | 5.4 ( 3.18 - 9.17 ) | 5.4 ( 49.1 ) | 5.3 ( 3.12 ) | 2.41 ( 1.29 ) | 7.19E-07 | 1.06E-04 |
| Antipsychotic | lurasidone | 6 | 12.21 ( 5.47 - 27.3 ) | 12.21 ( 61.18 ) | 12.11 ( 5.42 ) | 3.6 ( 1.13 ) | 1.32E-05 | 0.001942712 |
| Anxiolytic | clonazepam | 5 | 7.72 ( 3.2 - 18.6 ) | 7.71 ( 28.99 ) | 7.66 ( 3.18 ) | 2.94 ( 0.68 ) | 5.68E-04 | 0.083499959 |
| Central nervous system depressant | oxybate sodium | 81 | 39.23 ( 31.07 - 49.53 ) | 39.19 ( 2634.59 ) | 34.38 ( 27.23 ) | 5.1 ( 4.27 ) | 1.57E-94 | 2.32E-92 |
| CFTR modulator | elexacaftor ivacaftor tezacaftor | 4 | 9.57 ( 3.58 - 25.58 ) | 9.57 ( 30.49 ) | 9.51 ( 3.56 ) | 3.25 ( 0.52 ) | 9.26E-04 | 0.136053554 |
| Central nervous system stimulant | lisdexamfetamine | 5 | 8.05 ( 3.34 - 19.41 ) | 8.05 ( 30.63 ) | 7.99 ( 3.32 ) | 3 ( 0.7 ) | 4.70E-04 | 0.069055307 |
| Contraceptive | levonorgestrel | 43 | 7.08 ( 5.2 - 9.65 ) | 7.08 ( 209.49 ) | 6.67 ( 4.9 ) | 2.74 ( 2.11 ) | 3.10E-46 | 4.56E-44 |
| Contraceptive | etonogestrel | 18 | 10.16 ( 6.36 - 16.24 ) | 10.16 ( 144.49 ) | 9.9 ( 6.2 ) | 3.31 ( 2.08 ) | 1.08E-12 | 1.59E-10 |
| Contraceptive | ethinylestradiol etonogestrel | 15 | 24.02 ( 14.39 - 40.1 ) | 24 ( 322.98 ) | 23.47 ( 14.06 ) | 4.55 ( 2.56 ) | 4.37E-16 | 6.42E-14 |
| Contraceptive | intrauterine contraceptive device | 8 | 5.43 ( 2.7 - 10.9 ) | 5.43 ( 28.52 ) | 5.37 ( 2.67 ) | 2.43 ( 0.88 ) | 1.57E-04 | 0.023112969 |
| Contraceptive | drospirenone ethinylestradiol | 8 | 5.92 ( 2.95 - 11.89 ) | 5.92 ( 32.3 ) | 5.86 ( 2.92 ) | 2.55 ( 0.96 ) | 8.73E-05 | 0.01282664 |
| Contraceptive | ethinylestradiol levonorgestrel | 3 | 22.33 ( 7.18 - 69.44 ) | 22.31 ( 60.79 ) | 22.21 ( 7.14 ) | 4.47 ( 0.37 ) | 3.69E-04 | 0.054302527 |
| Immunomodulator | interferon beta 1a | 17 | 3.31 ( 2.04 - 5.36 ) | 3.31 ( 26.67 ) | 3.25 ( 2.01 ) | 1.7 ( 0.84 ) | 7.60E-07 | 1.12E-04 |
| Immunomodulator | certolizumab pegol | 23 | 8.08 ( 5.33 - 12.25 ) | 8.08 ( 137.59 ) | 7.83 ( 5.16 ) | 2.97 ( 2.01 ) | 1.00E-13 | 1.48E-11 |
| Opioid use disorder therapy | buprenorphine naloxone | 4 | 6.05 ( 2.27 - 16.18 ) | 6.05 ( 16.77 ) | 6.02 ( 2.25 ) | 2.59 ( 0.29 ) | 0.004764557 | 0.700389824 |
| Progestin | hydroxyprogesterone | 6 | 7.88 ( 3.53 - 17.61 ) | 7.88 ( 35.69 ) | 7.81 ( 3.5 ) | 2.97 ( 0.89 ) | 1.45E-04 | 0.021380096 |
| Uterotonic / Abortifacient | misoprostol | 4 | 57.33 ( 21.43 - 153.35 ) | 57.22 ( 219.58 ) | 56.87 ( 21.26 ) | 5.83 ( 0.93 ) | 9.53E-07 | 1.40E-04 |

PND, perinatal depression; FAERS, FDA Adverse Event Reporting System; ROR, reporting odds ratio; CI, confidence interval; PRR, proportional reporting ratio; χ^2^, chi-squared; BCPNN, Bayesian confidence propagation neural network; IC, information component; IC025, the lower limit of the 95% one-sided CI of the IC; EBGM: Empirical Bayes Geometric Mean; EBGM05, The lower limit of the 90% one-sided CI of the EBGM; P-adjust, p-value after Bonferroni correction; P-adjust<0.01, statistically significant.
